# Editing out HIV: application of gene editing technology to achieve functional cure

**DOI:** 10.1186/s12977-021-00581-1

**Published:** 2021-12-18

**Authors:** Jingna Xun, Xinyu Zhang, Shuyan Guo, Hongzhou Lu, Jun Chen

**Affiliations:** 1 Scientific Research Center, Shanghai Public Health Clinical Center, Fudan University, 2901 Caolang Road, Shanghai, 201508 China; 2 Shanghai Foreign Language School, Shanghai International Studies University, Shanghai, China; 3 Department of Infectious Diseases and Immunology, Shanghai Public Health Clinical Center, Fudan University, 2901 Caolang Road, Shanghai, 201508 China; 4 State Key Laboratory of Genetic Engineering, School of Life Sciences, Fudan University, Shanghai, China

**Keywords:** HIV/AIDS, Highly active antiretroviral therapy, Gene editing, Functional cure

## Abstract

Highly active antiretroviral therapy (HAART) successfully suppresses human immunodeficiency virus (HIV) replication and improves the quality of life of patients living with HIV. However, current HAART does not eradicate HIV infection because an HIV reservoir is established in latently infected cells and is not recognized by the immune system. The successful curative treatment of the Berlin and London patients following bone marrow transplantation inspired researchers to identify an approach for the functional cure of HIV. As a promising technology, gene editing-based strategies have attracted considerable attention and sparked much debate. Herein, we discuss the development of different gene editing strategies in the functional cure of HIV and highlight the potential for clinical applications prospects.

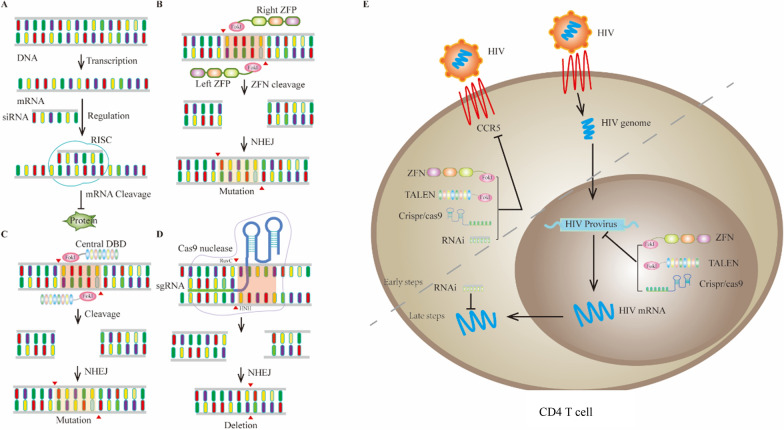

## Introduction

Human immunodeficiency virus (HIV) infection remains a major public health problem worldwide, with an estimated 38.0 million people living with HIV (PLWH) as of 2019, including 1.7 million newly infected patients [1]. Although highly active antiretroviral therapy (HAART) can suppress viral replication to undetectable levels and extend the life of PLWH, this approach does not offer a permanent cure because the HIV reservoir cannot be eradicated once it is established [2, 3]. The HIV-1 reservoir comprises full-length, replication-competent, but transcriptionally inactive virus [4]; once HAART is interrupted, this latent HIV reservoir can rebound [5]. Moreover, patients taking long-term HAART may experience major side effects, and the drugs can be expensive, limiting wide application and compliance [6]. Accordingly, achieving long-term host-mediated control of viral replication and remission of the symptoms of HIV-1 infection without long-term administration of HAART, i.e., functional cure of HIV, is an urgent priority in global HIV research.

In 2009, Hütter et al*.* [7] reported the “Berlin patient,” who had HIV and acute leukemia; after chemotherapy, radiotherapy, and myeloablative allogeneic hematopoietic stem cell transplant (HSCT), this patient was cured of leukemia, and the HIV viral load was undetectable. In a similar case (the “London patient”), the patient was a PLWH with Hodgkin’s lymphoma, and the functional cure of HIV was achieved after administration of HSCT [8]. Notably, both of these patients received HSCT from CCR5 Δ32 homozygous donors [9]. However, owing to the rarity of CCR5 Δ32 gene mutations and tropism changes in HIV strains after cell transplantation, this strategy is difficult to replicate and apply in clinical practice. Despite these limitations, gene editing strategies may be promising for achieving functional cure of HIV.

**Fig. 1:**
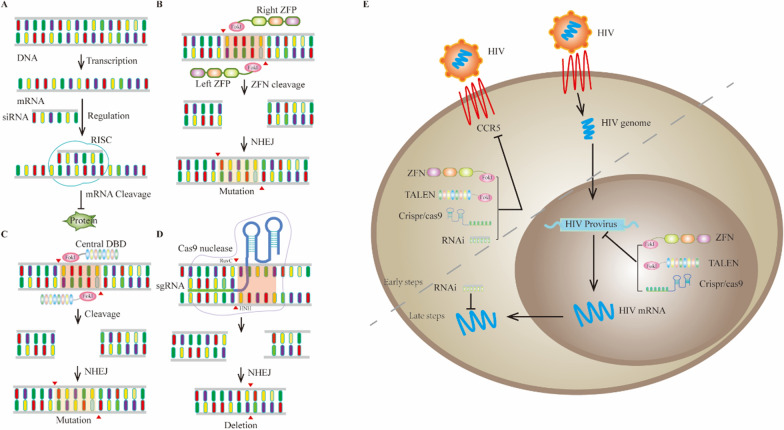
Schematic of different gene therapy strategies against HIV. **A** RNA interference (RNAi) processes the double stranded RNA (dsRNA) region of pathogenic RNA into small or short interfering RNAs (siRNAs), siRNAs bind to cell proteins to form RNA induced silencing complex (RISC), which can cleave foreign RNA sequences from siRNA. **B** Each ZFN consists of a cleavage domain of *Fok*I, which is fused with a zinc finger protein (ZFP), which has been customized with a gender specific “left” or “right” half site. The combination of two ZFNs makes the dimerization and DNA cleavage possible. **C** The TALEN elements are targeted to specific DNA sites by DNA recognition module, and then cleaved under the action of *Fok*I nuclease. **D** CRISPR/Cas system consists of CRISPR sequence elements and Cas gene family. The proteins encoded by these genes have the functional domain of nuclease activity, which can specifically cut the DNA sequence. **E** Application of different gene editing techniques in HIV eradication

Gene editing refers to the modification (e.g., deletion, insertion, frameshift mutation) of target genes by nuclease to alter gene function or phenotype. The gene editing techniques used in HIV therapy mainly include RNA interference [RNAi; small interfering RNA (siRNA) and short hairpin RNA (shRNA)]; programmable nuclease-based editing, such as zinc finger nucleases (ZFNs), transcription activator-like (TAL) effector nucleases (TALENs), and clustered regulatory interspaced short palindromic repeat (CRISPR; Fig. 1); and recombinant enzymes in vitro, which may be the most advanced systems currently available for inactivation or eradication of HIV genomes.

In this review, we summarize the most recent progress in different gene editing technologies and their applications in the functional cure of HIV.

## RNAi

RNAi is an endogenous cellular mechanism triggered by double-stranded RNA (dsRNA), which leads to the degradation of homologous RNAs [10]. When exogenous genes, such as viral genes, are randomly integrated into the host cell genome and transcribed, dsRNA is often generated [11, 12]. This dsRNA is recognized by an RNase type III enzyme, Dicer, and cleaved into siRNA, which can subsequently be unwound and assembled by certain enzymes (e.g., endonucleases, exonucleases, and helicases) to form effector complexes called RNA-induced silencing complexes (RISCs) [13, 14]. RISCs can cleave the sequence of the foreign RNA from which the siRNA was derived, thereby preventing translation [15]. Generally, under the control of Pol III promoters, siRNAs can also be expressed in cells using a DNA template to transcribe shRNAs [16, 17].

RNA-based therapeutic approaches are quickly emerging as adjunctive treatment methods for controlling HIV (Table 1). In 2001, Elbashir et al. [18] transfected cells with siRNA to selectively silence corresponding genes in mammalian cells. The findings of their study provided new tools for exploring gene functions of HIV in mammalian cells and for gene-specific therapeutics. Additionally, Park et al. [19] designed six long dsRNAs containing the HIV-1 *gag* and *env* genes to study RNAi-mediated gene editing in HIV-1-infected cells; they found that all of these dsRNAs could suppress HIV-1 replication. Chao et al. [20] also demonstrated that HIV-1-enhanced long noncoding RNA silencing using RNAi prevented HIV recrudescence in T cells and microglia upon cessation of azidothymidine treatment in vitro.

**Table 1:** Overview of past and current investigations by RNAi in HIV therapy

Years	Target	Cell types/organisms	References
2015	CCR5 and six regions in the viral genome	Hu-PBL mouse model	Choi et al. [96]
2018	The 5′ long terminal repeat (LTR)	Infected T lymphoblastoid CEM cell line and primary human CD4+ T-cells	Zhou et al. [23]
2019	HIV-1 Tat subtypes	HEK293T cells and TZM-bl cells	Ronsard et al. [97]
2019	HIV-1-enhanced lncRNA (HEAL)	MT4 and H9 cells and E4 Jurkat cells	Chao et al. [20]
2020	EC-LTNP or LTNP	Peripheral blood mononuclear cells (PBMCs)	Ayala-Suarez et al. [98]
2020	Long terminal repeat indexing-mediated integration site sequencing (LTRi-Seq)	Human CD34+ HSPCs and human fetal thymus and fetal livers	Suryawanshi et al. [27]
2020	NOP2	HIV-infected T cell line J89GFP and THP89GFP HIV-1 latency cell lines CD4+ T cells MAGI-HeLa, TZM-bl, and HEK293T fembryonic kidney cells	Kong et al. [99]

Because RNAi has become more widely used in studies of HIV infection, increasing numbers of siRNAs and shRNAs are being tested against HIV-1. For example, Lee et al. [21] detected three shRNAs that targeted three viral sites (*rev*, *gag*, and *vif*) and found that all three of the shRNAs inhibited the replication of the homologous HIV_IIIB_ strain. Novina et al. [22] also showed that siRNAs could inhibit virus production by targeting the mRNAs of the HIV-1 cellular receptor CD4, the viral structural Gag protein, or green fluorescent protein substituted for the Nef regulatory protein.

Notably, a small antisense RNA that targets a conserved region within the HIV-1 long terminal repeat (LTR) can remodel the surrounding chromatin by increasing both histone and DNA methylation and causing loss of nuclear factor-κB recruitment in primary human CD4^+^ T cells [23]. Furthermore, Turner et al. [24] used a clinically validated lentiviral vector termed HIV7-IGFP and found that LTR-362as could reduce virus replication in cell culture and primary human CD4^+^ T cells in a dose-dependent manner. Subsequently, the “sh1005/sh516” combination vector, which expressed two anti-HIV shRNAs, i.e., one directed at the HIV coreceptor CCR5 (sh1005) and the other directed at the LTR R region of HIV-1, showed antiviral efficacy against both R5- and X4-tropic HIV-1 in hematopoietic stem/progenitor cells (HSPCs) in a humanized bone marrow/liver/thymus mouse model [25–27]. Transplantation of sh1005/sh516-transduced HSPCs resulted in stable marking in hematopoietic lineages and potent inhibition of HIV-1-mediated depletion of modified CD4^+^ T cells in vivo [28].

Combinations of unmodified and genetically modified cells have been explored in clinical trials [29]. In a study of HSPC-based gene therapy, four patients with acquired immunodeficiency syndrome (AIDS)-related lymphoma were given gene-modified HSPCs transduced with a lentiviral vector encoding three RNA-based anti-HIV-1 moieties (tat/rev shRNA, TAR decoy, and CCR5 ribozyme) in addition to unmodified cells [30]. The treatment was well tolerated, and persistent expression of the introduced siRNA was observed; however, the anti-HIV-1 efficacy could not be determined.

Small RNAs that use RNAi pathways to target HIV-1 have been shown to be successful at inhibiting virus replication and new cell infection in vitro and have been included in all gene combinations that have entered clinical trials to date [31]. Nevertheless, the clinical application of siRNAs has been hindered by their limited cellular uptake and low biological stability, and the use of siRNA nanocarriers may be essential to overcome these barriers [32, 33]. Moreover, HIV-1 can also develop resistance to small RNAs [34]. If further developments in RNAi technology are able to overcome these limitations, curative treatment of HIV may be possible.

## ZFNs/TALENs

ZFNs are engineered restriction endonucleases designed to target specific DNA sequences within the genome [35]. Normally, ZFNs are made up of two functional domain modules composed of a programmable zinc-finger array and the nuclease domain of FokI, which are linked together by a linker peptide [36]. The DNA-binding domain and the cleavage domain of the type IIS FokI restriction endonuclease function independently of each other [37]. The zinc finger domain is a common DNA-binding domain encoded in the human genome that binds to DNA in a modular fashion [38]. Additionally, artificial nucleases can generate site-specific double-strand breaks (DSBs) within the genome predominantly by the error-prone nonhomologous end-joining (NHEJ) or homology-directed repair (HDR) pathway to promote genome editing [39]. TALENs are similar to ZFNs owing to the presence of amino acid repeats that are capable of binding DNA and the use of Fok1 as a nuclease effector [40].

To date, numerous studies have evaluated the application of ZFNs and TALENs to edit *CCR5* and other genes in an attempt to halt HIV-1 infection (Table 2). In these studies, researchers have used NHEJ to knockout genes in ex vivo autologous cell therapy, and somatic cells are then isolated, modified, and introduced back into the body [41]. Holt et al. used engineered ZFNs to disrupt the *CCR5* gene in human HSCs at a mean frequency of 17% of total alleles in a population and demonstrated retention of the ability to engraft NOD/SCID/IL2rγ-null mice (an effective HIV/AIDS model) [42, 43]. Mice transplanted with ZFN-modified HSCs received a rapid selection of CCR5-negative cells and were shown to have significantly lower HIV-1 levels with preservation of human cells throughout their tissues [42]. In another study, Perez et al. [44] used engineered ZFNs to disrupt endogenous CCR5; transient expression of CCR5 ZFN permanently and specifically destroyed approximately 50% of the CCR5 alleles in primary human CD4^+^ T cells. HIV-1-infected mice transplanted with ZFN-modified CD4^+^ T cells were then found to have lower viral loads and higher CD4^+^ T cell counts than mice transplanted with wild-type CD4^+^ T cells [44]. The same approach has been successfully applied to edit C-X-C chemokine receptor 4 (CXCR4). ZFN-modification of CXCR4 in CD4^+^ T cells was found to be stable, and HIV-1-infected mice transplanted with CXCR4 ZFN-modified CD4^+^ T cells showed lower viral loads than mice transplanted with unmodified CD4^+^ T cells [45].

**Table 2:** Overview of past and current investigations by ZFN or TALEN in HIV therapy

Years	Target	Cell types/organisms	References
2013	Endogenous CCR5 gene	HeLa cells	Ru et al. [100]
2014	CCR5 locus	Human CD4+ T cells	Yi et al. [101]
2014	LEDGF/p75 gene, PSIP1	HT1080, 293T, and Jurkat E6 cells	Fadel et al. [102]
2014	CCR5	The CCR5-1-GHOST cell line	Mock et al. [103]
2014	HIV-1 sub-type B DNA sequences	HeLa-tat-III/LTR/d1EGFP cells	Strong et al. [104]
2015	CCR5	MSC	Manotham et al. [105]
2015	CCR5	The T-cell line PM1	Mock et al. [54]
2016	CCR5	Human hematopoietic stem/progenitor cells	DiGiusto et al. [106]
2018	The third exon of CCR5	Nucleated CD34+ cells	Chattong et al. [107]
2018	Human CCR5 gene	HeLa cells or HEK293T cells	Liu et al. [108]
2018	Long terminal repeats (LTRs)	HEK293T cells	Ji et al. [109]
2018	CCR5	HEK293T cells	Nerys-Junior et al. [110]

This approach has also been tested in clinical trials. Tebas et al. recruited 12 patients who had chronic aviremic HIV infection and infused these patients with autologous CD4-enriched T cells modified at the *CCR5* gene locus using ZFNs. During the period of HAART interruption, the decrease in circulating CCR5-modified cells (− 1.81 cells/day) was significantly lower than that of unmodified cells (− 7.25 cells/day). In addition, the blood level of HIV DNA decreased in most patients. However, they observed serious adverse events related to the infusion of ZFN-modified autologous CD4^+^ T cells, which were attributed to a transfusion reaction [46]. The above-mentioned CCR5-specific ZFN developed by Sangamo BioSciences was tested in a phase I clinical study using a recombinant adenoviral vector for delivery (ClinicalTrials.gov number NCT00842634). However, the limited number of DNA targets available owing to restricted binding of zinc finger protein and the cytotoxicity caused by off-target cleavage hinder the development of ZFN-mediated therapies [47].

TALENs represent second-generation designer nucleases that significantly reduce off-target effects and thereby decrease cytotoxicity compared with ZFNs [48]. In a recent study, Shi used sequence analysis of polymerase chain reaction amplicons expressing the target regions for TALENs and ZFNs 48 h post-transfection, showing significant mutations in the CCR2 region of ZFN-treated cells, which had high homology with CCR5, but no mutations in the TALEN-treated cell population [49]. In contrast to ZFNs, TALEN delivery is often achieved using selected recombinant viral vectors, such as adenoviral vectors, adeno-associated virus (AAV) vectors, and lentiviral vectors, for in vivo experiments [50]. AAV-mediated delivery of TALENs and mega TALs (fusion of the TALE binding domain and mega nuclease cleavage domain) was found to enable editing of the *CCR5* gene in primary human T cells [51, 52]. However, the adenoviral and lentiviral plasmid vectors harboring TALEN sequences can be easily rearranged after transduction [53]. In addition, few viral vectors have been developed for HIV TALEN transgenes, and further research is still needed [50]. In later studies, Mock et al*.* introduced a new type of TALEN that can be effectively introduced into T cells through mRNA electroporation (a transient gene transfer technology). Their results showed that this approach resulted in highly efficient knockout of *CCR5* (> 90% in PM1 T cells and > 50% in primary T cells) [54]. Nevertheless, further studies are needed to evaluate the potential applications of TALENs in the functional cure of HIV, and the production of TALENs seems to be more challenging than that of ZFNs [55].

## CRISPR/Cas9

CRISPR tools are derived from an adaptive defense system found in most bacteria. The bacterial CRISPR/Cas9 system is composed of two elements: the nuclease protein Cas9, which cuts double-stranded DNA, and a single guide RNA (sgRNA) molecule that guides the Cas9 protein to a specific DNA sequence [56]. After cutting the double-strand DNA open, repair can occur through two basic mechanisms, i.e., NHEJ, a mechanism that allows the cell to randomly insert or delete nucleotides at the break site, and HDR, a mechanism that enables insertion of a template DNA to correct mutations at the DNA break site [57, 58]. CRISPR/cas9-induced DSBs are mainly repaired by NHEJ mechanisms [59].

Ebina et al. [60] successfully suppressed HIV-1 gene expression in Jurkat cells by targeting HIV-1 LTR with CRISPR/cas9 for the first time in 2013. Subsequently, the CRISPR/cas9 system has been used in the exploration of HIV treatments (Table 3). For example, Hu et al. [61] found that the CRISPR/Cas9 system can be used to identify the specific targets of complete excision and integration of the pre-HIV genome, leading to inactivation of viral gene expression and replication in HIV latently infected cells; which is a potential therapeutic advance in eliminating barriers of all pro-viruses in HIV-1 infected people. In addition to the HIV provirus, other researchers have focused on the HIV receptor. Indeed, Wang et al. [62] used a lentivirus expressing CCR5-sgRNA and Cas9 to knockout the coreceptor CCR5 in CD4^+^ T cells, making them resistant to HIV-1. Two different gRNA combinations targeting both *CXCR4* and *CCR5* were designed by Guo’s team. The CRISPR-sgRNA-Cas9 system successfully induced *CXCR4* and *CCR5* gene editing in various cell lines and primary CD4^+^ T cells, indicating that this CRISPR/Cas9 approach could have applications in the functional cure of HIV/AIDS [63].

**Table 3:** Overview of past and current investigations by CRISPR/Cas9 in HIV therapy

Years	Target region	Cell type/organism	References
2014	HIV-1 LTR promoter U3 region	Myeloid lineage cells	Hu et al. [61]
2015	CCR5 locus	Primary human T cells	Sather et al. [52]
2016	Heteroduplex of wild type and mutant CCR5 delta 32 (i) Middle band (ii) Upper band (iii) Lower band	(i) Human embryonic kidney HEK 293T cells (ii) Human acute T cell (iii) Leukemia cell line (iv) Human breast adenocarcinoma cell line MDA-MB-231 cells	Qi et al. [111]
2017	Gene KO in both mouse and human T cells	CD4+ and CD8+ T cells from mouse and human	Seki et al. [112]
2017	GPI-scFv X5	CD4 cells in hu-PBL mice sand mice with GPI-scFv AB65-transduction	Ye et al. [113]
2017	Human CCR5 locus in peripheral lymphocytes from long-term reconstituted mice	Human CD34+ cells	Xu et al. [66]
2017	Four different sites of the HIV-1 long terminal repeat (LTR)	HEK293T cells in humanized Bone marrow/liver/thymus (BLT) mice with chronic HIV-1 infection	Yin et al. [65]
2017	Gene correction and the knock-in of reporter genes into the rat nestin and human DARPP-32 genes	Human embryonic kidney (HEK) 293T and U2OS cells, C6 cells and human adult dermal fibroblasts	Gaj et al. [114]
2017	Genetic disruption of Pdcd1 in CAR T cells	Primary human T cells Purified human CD4+ or CD8+ T cells	Rupp et al. [115]
2017	Pcsk9 native, 5′ and 3′ and e-sgRNAs targeting mouse fumarylacetoacetate hydrolase (Fah) and ROSA26 loci	HEK293 cells in human	Yin et al. [116]
2019	Short single-stranded DNA HDR donor	Mammalian cells: CD34+ HSPCs	Wu et al. [117]

Using two transgenic mouse models, Kaminski injected plasmid vectors expressing cas9 and various gRNAs into the tail vein or peritoneum. A large basic HIV DNA fragment was excised from the HIV-1 provirus and then detected in the spleen, liver, heart, lungs, and lymphocytes of mice, indicating that integrated HIV-1 provirus could be eliminated in vivo using CRISPR/cas9 in many different cells and tissues [64]. Furthermore, Yin et al. [65] demonstrated the feasibility and efficiency of this approach using AAV combined with multiple sgRNAs and *Staphylococcus aureus* Cas9 to destroy HIV-1 provirus in three different animal models. These findings established a foundation for the design of clinical trials in humans.

Based on these data showing that CRISPR/Cas9 can be used to edit the pro-HIV genome or CCR5 receptor in vivo and animal models, dsRNA are now attempting to achieve autologous HSCT through gene editing technology in clinical trials. For example, Deng et al. [66] successfully established a CRISPR/Cas9-mediated CCR5 ablating system in long-term HSCs and showed that this system conferred HIV-1 resistance in vivo. Subsequently, the team reported the first case of successful allogeneic transplantation and long-term engraftment of CRISPR/Cas9-edited HSPCs to a patient with HIV and acute leukemia. The patient’s symptoms of leukemia were then reported to be in complete remission [67], demonstrating that long-term persistence of CRISPR-edited allogeneic HSPCs is possible. However, NHEJ repair is error prone and introduces short insertions and deletions (indels), which remain after cas9/sgRNA cleavage and often interfere with the function of the target DNA [68]. Most of these indels are indeed lethal to HIV-1, although some indels have been shown to lead to the emergence of replication-active HIV-1 resistant to cas9/sgRNA [68, 69]. This resistance may accelerate the escape of HIV-1, which could limit the application of cas9/sgRNA in HIV-1 treatment [70].

## In vitro-engineered recombinase

Although the CRISPR/cas9 system has some advantages over other technologies, it can cause unpredictable damage via the DNA repair mechanism and can result in virus escape. As an alternative, HIV genome editing may be achieved using engineered recombinase enzymes [71]. As a novel gene editing technology that can safely remove HIV provirus from cells, LTR-specific recombinase (TRE recombinase) was recently reported [72] and may represent a new strategy for HIV eradication. TRE, an engineered version of cyclization recombination enzyme (Cre) recombinase, was designed to target a 34-bp sequence within the HIV-1 LTR (*loxLTR*) sequence [73]. Expression of TRE in HIV-1-infected cells containing *loxLTR* sequences results in the removal of the integrated proviral DNA in infected cultured cells [73]. Moreover, TRE-mediated antiviral effects have been demonstrated in TRE-transduced primary CD4^+^ T cells or TRE-transduced CD34^+^ HSCs in HIV-infected humanized RAG2^−/−^ γC^−/−^ mice [74].

Because the *loxLTR* sequence is not highly conserved among different HIV-1 subtypes, it is not suitable as a target for eradication of provirus from most HIV-1-infected individuals. Therefore, Karpinski et al. reported the development and application of broad-spectrum recombinase 1 (brec1), which shows activity against most primary HIV-1 isolates. Brec1 can specifically recognize and recombine a highly conserved target site (*loxBTR*) located in the LTR sequence of most HIV-1 isolates to remove provirus from HIV-1-infected cells. Indeed, more than 72% of HIV-infected individuals worldwide have HIV-1 subtypes M, A, B, or C, and 90% of these patients are expected to harbor the exact *loxbtr* sequence targeted by Brec1 [75]. Brec1 is derived from the mature Cre/loxP system, which facilitates directed evolution by substrate linkage. The engineered recombinase has significant advantages over traditional knockouts (e.g., ZFNs, TALENs, and CRISPR/Cas9), for which gene deletion and off-target effects can be lethal. By contrast, engineered recombinases are independent of cellular pathways and do not activate DNA repair pathways during genome editing. Nevertheless, the safety of engineered recombinases and their side effects in edited cells still need to be evaluated.

## Discussion and perspective

When HIV infects a new host, it spreads to the lymph nodes and blood within 1–2 weeks. During this process, the HIV reservoir is established throughout the whole body, including the central nervous system, lymphoid tissue (i.e., the spleen, thymus, lymph nodes, and intestinal-related lymphoid tissue), bone marrow, lungs, kidneys, liver, adipose tissue, gastrointestinal tract, and urogenital system [76–79]. The lymph nodes are the main reservoir, with a large number of target cells, high level of activation, and high level of replication, resulting in infection of new cells [80]. Gut-associated lymphoid tissue (GALT) contains 60% of human lymphocytes and plays important roles in the pathogenesis of HIV infection through Th17 cell depletion, bacterial translocation, and local host cell activation [81–84]. The main reservoirs are resting CD4^+^ T cells [85]. In addition, other cell types are known to be infected with HIV, establishing the HIV reservoir. For example, macrophages, microglia, and astrocytes in the central nervous system may be infected with HIV, and microglia are considered the main reservoir in the brain [86, 87]. The effectiveness of antiretroviral drugs is anatomically and pharmacologically limited in the brain region, contributing to the persistence of the virus in the brain [88, 89].

There is no standard method to test the HIV reservoir. Some studies have characterized the reservoir based on the level of total HIV DNA [80, 90, 91]. Interestingly, during acute and early HIV infection, gastrointestinal CD4^+^ T cells have been shown to contain 13-fold higher levels of HIV DNA than blood CD4^+^ T cells [92]. Additionally, in the intestinal tract, non-CD4 T cells contain less HIV DNA than CD4^+^ T cells; however, the infection level of non-T leukocytes in the GALT is higher than that in the blood [93]. Although HAART can quickly maintain the viral load at a very low level when activated during acute and chronic infection, the continuous transcription of viral RNA can still be detected in lymphoid tissue at the main site of viral transcription [94]. Therefore, strategies aiming to clear the HIV reservoir are essential for achieving functional cure of HIV. As described in this review, targeted knockout of HIV integration fragments based on gene editing technology prevents the transcription and translation of HIV, thereby suppressing the formation of new virus particles. However, the biggest problem with gene editing technology is efficiency. Whether higher editing efficiency means greater risk of side effects remains unclear, and further studies are needed to verify the effectiveness and safety of these methods.

The editing efficacy and duration of editing effects, particularly in vivo, still warrant improvement, and the safety and off-target effects of these approaches are concerning. Therefore, gene editing technologies are the subject of many ethical discussions. After these disadvantages are overcome, gene editing technology is expected to be a promising approach, particularly when used in combination with other therapies that affect HIV replication. For example, chimeric antigen receptor (CAR) T-cell (CAR-T) therapy, as a new tool to target the HIV reservoir, may be combined with gene editing technology to promote immune system function, and this safe approach may be an effective strategy for achieving functional cure of HIV. Moreover, the combination of autologous cell gene editing and stem cell transplantation could present rejection related to allogeneic stem cell transplantation, and CRISPR/Cas9 combined with HAART can promote the so-called “shock and kill” strategy [95]. Taken together, these studies indicate that gene editing technology combined with other treatment strategies may be an effective approach for achieving functional cure of HIV.

## Conclusion

In summary, gene editing technology has improved our understanding of the interactions between the host and virus, enabling the creation of new animal models of HIV infection and providing new strategies for the functional cure of HIV.

## Data Availability

Not applicable.
